# Liquid Trisilanol i-Octyl POSS Achieves Rapid Hemostasis and Pneumostasis in Experimental Lung Injury

**DOI:** 10.3390/pathophysiology33010001

**Published:** 2025-12-22

**Authors:** Michelle Tucci, Robert C. O′Brien, Joseph D. Lichtenhan, Hamed Benghuzzi, Drew Hildebrandt

**Affiliations:** 1 Anesthesiology, University of Mississippi Medical Center (UMMC), Jackson, MS 39216, USA; 2 Ophthalmology, Miller School of Medicine, University of Miami, Miami, FL 33146, USA; robrien@med.miami.edu; 3 Hybrid Plastics, Inc., Hattiesburg, MS 39401, USA; lichtenhan@hybridplastics.com; 4 Biology, Jackson State University, Jackson, MS 39217, USA; hamed.a.benghuzzi@jsums.edu; 5 Surgery, University of Mississippi Medical Center, Jackson, MS 39216, USA; dhildebrandt@umc.edu

**Keywords:** trimethylpentyl polysilsesquioxane, POSS, hemostasis, chitin, kaolin, noncompressive

## Abstract

**Background/Objectives**: No effective intervention currently exists for non-compressible pulmonary injury, especially in a prehospital setting. Visco-liquids like trisilanol i-octyl POSS could remedy this. POSS resists hemorrhage and activates clotting; this can be augmented with kaolin (22.5%; PK) or chitin (10%; PC). **Methods**: We tested the efficacy of POSS, PK, and PC in treating incisional lung wounds in swine (39 ± 1 kg; *n* = 10). An incisional wound was made in the lung via a left thoracotomy, allowed to bleed freely for 30 s, and then no treatment (UNT), gauze with compression (GC), or POSS, PK, or PC was applied (1.5 mL). Each treatment was applied once per animal for a total of 5 wounds. Wounds were observed for 10 min for hemostasis and pneumostasis; GC treatments were assessed at 3 min intervals. **Results**: POSS and PC produced hemostasis in 8 of 10 wounds; GC: 7 (all significant from UNT); PK: 5 and UNT: 1. PK was not different from any group. POSS (2 ± 0.3 min) and PC (1.4 ± 0.4 min) clotted more quickly than GC (8 ± 3 min); PK was intermediate (3.8 ± 2 min) and not different from any other group. Pneumostasis was achieved in all POSS, PC, and PK, and only after hemostasis in the GC group. **Conclusions**: Because both POSS and PC provided quick and lasting hemorrhage and pneumatic control in this model, without need for compression, these results support the concept that these types of liquid POSS compounds could prove to be efficacious in prehospital treatment of non-compressible trauma wounds.

## 1. Introduction

Thoracic organ hemorrhage is difficult to control, not only in a hospital setting, but especially so by first responders on the battlefield and in civilian trauma, which can result in increased mortality and morbidity. For example, a study of battlefield injuries in Iraq and Afghanistan showed that 91% of survivable mortality was due to uncontrolled hemorrhage; 62% of this was non-compressible hemorrhage in either the thoracic or abdominal cavity [1].

To stop bleeding in a controlled, clinical setting, a number of options are available, and if needed, the lung can be accessed surgically and pressure applied to the wound, with or without a hemostatic agent [2]. Of course, first responders normally do not have this choice. For wounds in general, first responders normally apply a bandage and then pressure, usually by direct compression of the wound [3,4]. Although devices (e.g., tourniquets, pelvic binders, and REBOA) are available to aid in wound compression from peripheral and even lower abdominal sites, no such equipment is available that works on thoracic organ hemorrhage, or internal organ hemorrhage in general, in a non-hospital setting. The standard treatment, then, is to pack the wound with gauze with or without a hemostatic agent, apply direct pressure as much as possible, and transport to a medical facility where additional options exist for managing the bleeding [3,4]. Not only is this not always effective, but success is also dependent on rapid evacuation of the patient to a medical facility; this option is not always available. Moreover, this treatment option is not very effective for thoracic cavity wounds because of the presence of the rigid rib cage. Thus, although a number of treatments have been devised, currently it can be said that there is no technology that is singularly effective against hemorrhage associated with injured lungs, where air loss concomitant with bleeding may add an additional component to the situation, especially in prehospital situations. In this case, effective treatment would produce a physical seal against air loss while also slowing or arresting blood loss.

Visco-liquids with hemostatic and self-sealing properties could be a readily deployable alternative to the current process of treating the hemorrhage and air loss. Liquid hemostatic sealants are not yet a common form of treatment despite the high propensity of a liquid to be proliferative within a wound channel and therefore of great potential utility in treating complex and difficult-to-access internal bleeds.

One such visco-liquid sealant with hemostatic properties is based on Polyhedral Oligomeric Silsesquioxanes (POSS), which are nanoscale hybrid molecules with the empirical formula (RSiO_1.5_)*_n_*, where *R* represents an organic substituent and *n* commonly equals 8, forming a T_8_ cubic cage structure [5,6,7]. Each POSS unit consists of an inorganic siloxane (Si–O–Si) framework with covalently attached organic functional groups at the silicon vertices. When incorporated into polymer systems, POSS enhances mechanical strength and thermal stability due to its rigid siloxane cage and strong interfacial interactions within the host matrix [5,6]. Extensive in vitro and in vivo studies have demonstrated the biocompatibility of POSS-based polymers. POSS-containing materials exhibit low cytotoxicity in fibroblasts, osteoblasts, and endothelial cells and show excellent hemocompatibility, with PEG–POSS surfaces, reducing platelet adhesion and fibrin formation compared to conventional siloxanes [6]. In vivo, POSS-polycarbonate urethanes and POSS–silicones elicit minimal fibrotic capsule formation and favorable tissue integration [6,7]. The siloxane cage itself is chemically stable, while degradation primarily occurs through cleavage of organic linkages, allowing predictable erosion kinetics when integrated into degradable matrices. In surgical applications, POSS-modified coatings reduce foreign body response and fibrotic adhesion formation by limiting protein adsorption, macrophage activation, and fibroblast proliferation at the implant interface [7]. POSS molecules are available in multiple functional forms, enabling precise tailoring within biomedical polymers: monofunctional POSS (e.g., glycidyl or methacrylate POSS) can serve as pendant chain modifiers, whereas multifunctional POSS (e.g., octa-aminopropyl or octa-hydrido POSS) can act as crosslinking nodes or nanoscale reinforcement centers [5,7]. This versatility allows tunable control over coating adhesion, durability, drug or anti-inflammatory agent release, and surface morphology, all of which are critical to optimizing tissue compatibility and healing outcomes [6].

POSS has also been shown in vitro to initiate clotting [8] and to stabilize and strengthen clots, and in vivo to effectively control hemorrhage in a femoral arterial swine model [9]. POSS gels are viscous liquids that provide physical resistance to fluids (blood and potentially air) flowing from a wound in addition to activating the clotting cascade through their silanol (Si-OH) groups. The sealing and hemostatic properties can be augmented further by the addition of either kaolin (22.5%; PK) or chitin (10%; PC). Prior to this work, the effectiveness of POSS and its formulated derivatives in treating internal organ injuries has not been evaluated in vivo, especially in models of non-compressible lung hemorrhage injury; thus, this study is the first of its kind to do so.

Therefore, the aims of this study were 1: to evaluate the efficacy of POSS compounds in controlling hemorrhage and air leakage in lung injuries, compared with gauze plus compression, and 2: to compare the effectiveness in these actions among the three different POSS compounds.

## 2. Methods

### 2.1. Assurances

The animal study protocol was approved by the University of Mississippi Medical Center Institutional Animal Care and Use Committee and the United States Army Medical Research and Development Command Animal Care and Use Office (protocol code # 1514 and date of approval: 19 June 2020). The Association for Assessment and Accreditation of Laboratory Animal Care accredits the UMMC facilities where these experiments were conducted. In conducting this research, the investigators adhered to the Animal Welfare Act Regulations and other Federal statutes relating to animals and experiments involving animals and the principles set forth in the current version of the *Guide for the Care and Use of Laboratory Animals, National Research Council* [10].

### 2.2. Animal Preparation

Male adult swine 39 ± 1 kg (*n* = 10) were obtained from Valley Brook Farms, Madison, Georgia, and were held for a minimum of 5 days to allow for acclimatization and for observation by UMMC veterinarians. During this time, they had free access to food and water; they were denied food starting ~17 h prior to being anesthetized. After premedication with an intramuscular cocktail of atropine (0.025 mg/kg), dexmedetomidine (0.08 mg/kg), and butorphanol (0.2 mg/kg), anesthesia was induced with ketamine (10 mg/kg). Following orotracheal intubation, the pigs were ventilated to maintain a PO_2_ of at least 95% and an end-tidal CO_2_ of 40–45%. Initially, they were maintained on isoflurane at 3%; this was reduced to 2% following surgery and for the duration of the experiment. Both lungs were ventilated, which would increase the difficulty of treatment [3], but it is the situation one could face as a first responder. The left carotid artery and internal jugular vein were exposed via cutdown, and a Millar catheter (Millar, Inc., Houston, TX, USA) inserted into the artery for continuous measurement of MAP and HR using a PowerLab data acquisition system and LabChart software (ADInstruments, Inc., Colorado Springs, CO, USA). The jugular vein was cannulated for infusion of sterile isotonic saline at ~6 mL/kg/hour for the duration of the procedure.

A thoracotomy was then performed at the 4th intercostal space on the left side, the 5th rib was removed, and a rib spreader inserted and opened to provide unobstructed access to the lung. Throughout the experiment, the exposed lung was moistened with periodic superfusions of small amounts of isotonic saline.

Immediately following the completion of the surgery, hetastarch (Hextend; 500 mL) was given i.v. over 15 min to replace any fluid lost during the surgery and to help maintain fluid homeostasis.

### 2.3. POSS Polymer Gels

POSS was provided by Hybrid Plastics Inc. at a <99% devolatilized purity level and thermally sterilized by falling film distillation at 120 °C (@10^−6^ torr) over 4 h.

In order to increase coagulation ability and durability of the gel seal against displacement by blood, POSS was mixed with either kaolin (PK, 87.5:22.5 wt%) or chitin (PC, 90:10% wt%). Both substances were used as received from Sigma Aldrich (St. Louis, MO, USA). The mixing was accomplished using a high-shear mixer (Silverson, East Longmeadow, MA, USA; 5000 rpm, 5 min, 90 °C) until visual homogeneity and steady-state torque were observed.

### 2.4. Experimental Protocol

Following recovery from surgery and stabilization of measured variables, and 15 min of control data collection, a series of 5 transverse linear incisions (10 cm long × 3 mm deep, ~5 cm apart) was made sequentially with a number 11 scalpel blade, starting toward the distal end of the exposed lung and proceeding proximally, and treated as described below. It should be noted that the incisions were made during maximal expiration, when the lung was at its minimal inflation, and these incisions not only cut through a substantial portion of the thickness of the lung, but upon expansion, the incisions expanded as well. Thus, while these may seem like shallow, superficial wounds, they actually injured a substantial portion of that lung lobe.

The incision was allowed to bleed freely for 30 s and then either POSS, PK, or PC was applied to the site as a bolus (1.5 mL) with no backing, or either no treatment (UNT), or gauze applied with compression (GC) as controls. Each of the treatments was applied once and their order was randomized within a given animal (simple randomization using Microsoft^®^ Excel (Rand) function, not blinded; Excel Office 2019). Following treatment, the wounds were allowed to remain undisturbed for a total of 10 min and observed for rebleeding and air leakage, except that GC treatment was assessed at 3 min intervals for hemostasis and leakage status by gently removing the gauze; gauze and compression were reapplied to any incision still bleeding. Air loss from the lung wound was determined by watching for air bubbles forming in the wounds upon positive pressure ventilation at 20 cm H_2_O. It is recognized that this is a somewhat subjective determination and that subtle differences in the amount of air leaking could exist between the different treatments. However, it was beyond the scope of this study to make those measurements; we simply were trying to determine if an incision leaked air following treatment. The fact that we always had three observers making these judgements should mitigate to a large degree the subjective nature of the measurement. Gauze and compression were used to provide hemostasis and air seal in any wounds in which these were not realized at 10 min. After the 10 min period, and once control of these variables was achieved, another wound was made and treated in a similar manner using one of the other treatments. A total of 5 lung wounds were made in each of the 10 pigs, for 50 total wounds, or 10 incisions per treatment group.

### 2.5. Data Analysis

MAP and HR were sampled continuously at 100 Hz and then analyzed in 5 min blocks. The time to reach hemostasis was determined visually by two or three observers by constant examination of the wound for active bleeding and was calculated from when the time treatment was applied to when the bleeding initially stopped. Pneumostasis in the lung incisions was ascertained as occurring when no more air bubbles were observed coming from the wounds following treatment. At the end of the experiment, the thoracic cavity was filled with saline to confirm pneumostasis.

Two-way Analysis of Variance for Repeated Measures was used for initial analysis of the MAP and HR data, followed by Dunnett’s test for data within a group. Between-group data for each time point were analyzed with ANOVA followed by Tukey’s Multiple Comparison Test.

Wound clotting was analyzed using a generalized estimating equations logistic regression model to account for wounds clustered within pigs; these data were binary, simply “yes” or “no” regarding clotting. For assessing differences in time-to-clot among the four treatments (GC, POSS, PC, and PK) and no treatment (UNT), the mean differences were estimated using Tobit regression with robust standard errors to account for censoring of time-to-clot at ten minutes and clustering of time-to-clot measurements on the same pig. Tukey’s method was used to adjust for multiple comparisons.

The determination of pneumostasis during the experiment was difficult as it depended on having blood or a fluid layer over the incision; although we attempted to keep the lung bathed in a film of saline throughout the study, this was not always possible, nor possible to do so in a manner that was the same during all treatments, so these data are treated as qualitative, not quantitative, and were not analyzed statistically.

All statistical tests for MAP and HR were performed using GraphPad Prism software (Version 10; GraphPad Software, San Diego, CA, USA). Binary wound clotting and time-to-clot statistical analyses were performed using R version 5.0 with the tidyverse, haven, AER, geepack, and emmeans packages. Data are presented as mean ± SEM.

Data values were considered significantly different at a *p*-value of < 0.05.

## 3. Results

Compared with the pre-incisional control period, MAP did not change during the duration of the experiment; thus, MAP was the same for all the treatment groups. Furthermore, MAP remained well above the accepted 65 mm Hg arterial rebleed pressure in all animals (Table 1) [11]. Likewise, HR was also unchanged from pre-incisional control and not different among the groups, demonstrating that all the pigs were in a stable homeostatic condition during the experiment.

**Figure 1:**
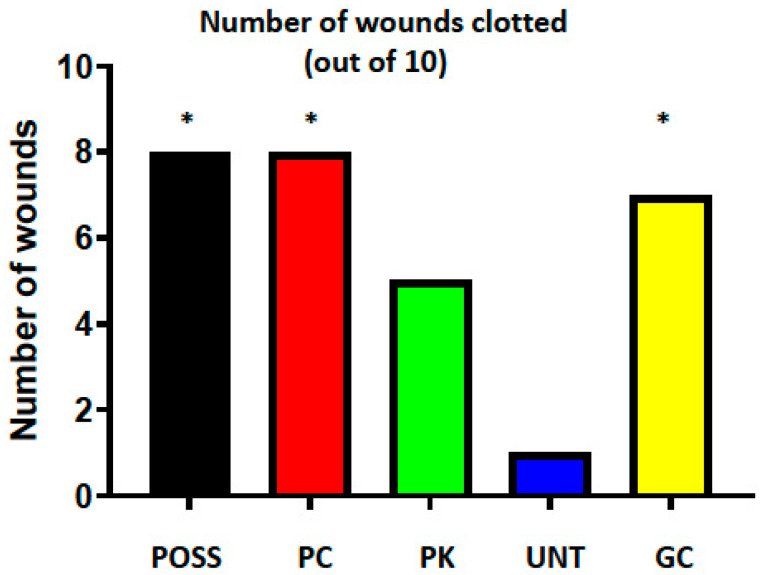
The number of incisional lung wounds (out of 10/group) that clotted within a 10 min period following treatment with polyhedral oligomeric silsesquioxane (POSS), POSS + chitin (PC, 10%), POSS + kaolin (PK, 22.5%), untreated (UNT), and gauze and compression (GC). * *p* < 0.05 compared with UNT; no significant differences among the other groups.

The viscous POSS formulation achieved hemostasis in 8 of 10 wounds, as did the PC (Figure 1). Seven wounds clotted in the GC group. None of these proportions were significantly different from each other, but POSS and PC were significantly greater than the UNT group, in which only one wound clotted (both *p* = 0.03). The PK group was intermediate between the UNT and the remaining groups (5 of 10 wounds clotted) and was not significantly different from any of the groups. None of the wounds rebled once hemostasis was achieved.

Table 2 shows the Tobit analysis in which there were statistically significant differences in minutes-to-clot between PC and UNT (estimated mean differences (EMD): 13.3 min; *p* < 0.001), PK and UNT (EMD: 8.4 min; *p* = 0.008), and POSS and UNT (EMD: 12.8 min; *p* = 0.001). Significant differences were also found between PC and GC (EMD: 8.3 min; *p* = 0.03), and POSS and GC (EMD: 7.7 min; *p* = 0.001), but there were no other statistically significant treatment differences.

**Figure 2:**
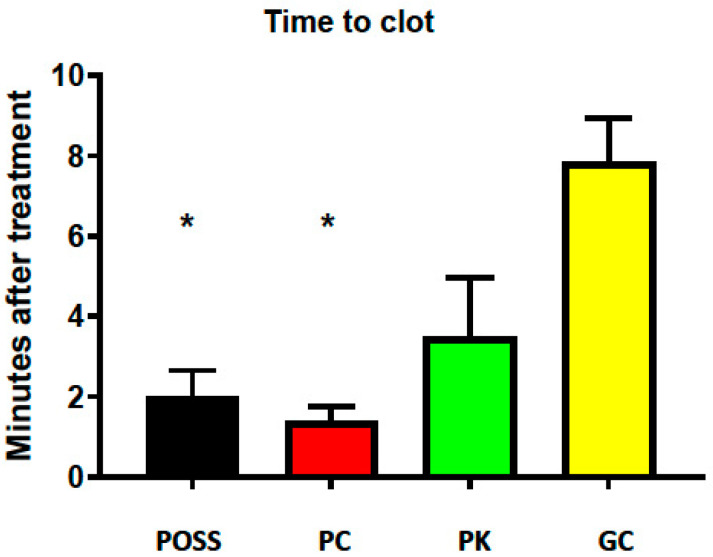
Time-to-clot following incisional wound treated with polyhedral oligomeric silsesquioxane (POSS), POSS + chitin (PC, 10%), POSS + kaolin (PK, 22.5%), or GC (gauze and compression). * *p* < 0.05 compared with GC.

Both POSS- (2 ± 0.3 min) and PC- (1.4 ± 0.4 min) treated groups clotted more quickly compared with the GC group (8 ± 3 min) (Figure 2). As before, the PK treatment effect was intermediate between GC and the other two POSS-treated incision groups and the time-to-clot (3.8 ± 2 min) was not significantly different from any of the other groups.

Pneumostasis was achieved in all the wounds treated with the three POSS formulations, even those that exhibited only partial hemostasis following treatment, but in the GC group only after hemostasis was achieved, and at no time in the UNT group. The strength of the seal against air loss also was verified post hoc by inflating the lungs to 20 cm H_2_O after filling the thoracic cavity with saline and watching for air leaks. None of the wounds that had achieved pneumostasis leaked air at this time.

## 4. Discussion

The present study demonstrates that both viscoelastic POSS and POSS–chitin formulations provide rapid, durable hemostasis and pneumostasis in a swine model of non-compressible pulmonary injury without the need for external compression. These findings support further development of POSS-based visco-liquid agents as potential field-deployable hemostatic and lung-sealing technologies, particularly in environments where rapid access to surgical intervention is limited. By extension, then, POSS should also prove useful for lung wound treatment in a clinical setting. This study adds to a growing body of literature evaluating next-generation hemostatic materials that function not only by accelerating coagulation but also by providing mechanical sealing of injured tissues [12,13,14,15,16].

Several aspects of POSS performance are notable. First, both POSS and PC provided comparable or superior hemostatic efficacy to standard gauze-and-compression treatment, despite being applied without any pressure. This highlights the utility of viscoelastic liquids capable of conforming to irregular wound geometries, an advantage shared with some emerging flowable hemostatic agents such as chitosan hydrogels [17,18], polyethylene glycol (PEG)based sealants [15,16,17,18,19,20,21,22], and fibrin-based foams [17]. Recent work on bioadhesive hydrogels [14,17,19,21] and biomimetic sealants such as mussel-inspired catechol polymers [15,18], polyurethane-urea sealants [23], and photopolymerizable lung patches [16] also underscores the increasing interest in therapies capable of withstanding respiratory motion and providing airtight seals in pulmonary applications.

Second, the reliable pneumostasis observed with all POSS formulations parallels outcomes reported for several novel lung sealants designed to treat alveolar air leakage (AAL). Studies have shown that nanotopographical silicone sealants [12,22], catechol-modified hydrogels [19], and polymer-reinforced fibrin patches [20] can reduce AAL in preclinical lung models by forming cohesive films that are resistant to shear deformation. POSS may have similar mechanical advantages due to its nanoscale siloxane cage structure, viscoelasticity, and hydrophobic interactions, which likely contribute to its ability to remain in place on a dynamic, low-adhesion lung surface.

Additional literature also supports the biocompatibility and wound-modulating properties of POSS. Prior investigations have reported minimal fibrosis, limited inflammatory capsule formation, and favorable tissue integration when POSS is incorporated into biomedical polymers. These attributes are particularly valuable for thoracic and pulmonary applications where excessive fibrosis or adhesions can impair lung expansion, impede healing, or complicate future surgical procedures.

Nonetheless, several limitations of the present work warrant further investigation. The model evaluated only moderate incisional parenchymal wounds; whether POSS can control bleeding from larger pulmonary arteries or high-pressure bronchial vessels remains to be tested. To this end, though, we have previously demonstrated that POSS, PC, and PK can stop or substantially slow hemorrhage from junctional arterial wounds [9], and there is no reason to suspect that they would not perform as well with larger vessel thoracic hemorrhage.

Moreover, the open thoracotomy approach, while necessary to control experimental variables, does not fully replicate the challenges of treating deep or inaccessible thoracic wounds encountered in field or civilian trauma. We did so for three reasons. First, this approach allowed us to use only 10 rather than 50 animals, which would have been the case had we used a closed thoracic cavity approach. Secondly, we needed to determine if the visco-elastic POSS compounds would stay in place on the wound or slide off; it is notoriously difficult to make substances adhere to lung surfaces, which by necessity are anti-adhesive. Finally, it was important to have such access to be able to control the amount and placement of wound treatments to cover the wound while avoiding excess spill-over to adjacent tissue, thus allowing multiple wounds per lung, and to easily determine if and when the treatments were effective. In a real-life situation, one simply would place the POSS-delivery device (e.g., syringe or Doypack) in the wound opening and infuse POSS in whatever amount is needed for success; there is no need to visualize the actual wounded tissue. The patient can then be moved without need for packing because we have shown previously that vigorous movement did not dislodge the clot from an excisional wound in a femoral artery or cause rebleeding [11]. Future studies are planned in which the wounding and treatment with POSS are performed with minimal thoracic access.

We examined only the acute actions of POSS compounds in achieving hemostasis; how long this hemostasis can last without addition intervention needs to be addressed in additional studies, as does the question of whether POSS treatment has deleterious chronic effects on pulmonary tissue. These questions will be addressed in future studies in which wounds are made and treated under asceptic techniques and then the animals allowed to recover while being monitored over time for indications of distress (behavioral changes, appetite, etc.). These animals would then be sacrificed at various time points and, not only would gross examination of the chest cavity and wound site be made, but also histological preparations made and examined to assess the extent of structural injury, inflammation, and repair. Key findings would include alveolar wall disruption, interstitial and subpleural emphysema, hemorrhage, and inflammatory infiltration by neutrophils and macrophages. Evidence of tissue repair would be marked by fibroblast proliferation, type II pneumocyte hyperplasia, and collagen deposition.

POSS is not an adhesive, thus is removed easily from a wound by wiping with gauze, sometimes with 70% ethanol applied to the gauze pre-wipe; [24] does not migrate from the compartment in which it has been placed; [25,26] and has been shown to have no harmful effects on tissue such as granulomas or increased tissue adhesion, with either short or long-term exposure [24,25,26]. In fact, we have shown that POSS compounds actually protect wounds from becoming infected and enhance healing [24]. However, long-term healing responses, durability of pneumostasis and hemostasis under physiologic movement, and potential interactions with infected or contaminated wounds warrant future controlled survival studies.

The observers were not blinded to the treatment group, which they normally would be. This was not possible because each treatment type had a unique appearance, so there was no way to blind the observers. However, we do not feel this is a major issue because the assessment of bleeding was not a nuanced decision of the degree of bleeding, but rather a clear binary judgment—“yes” or “no”.

Another possible limitation is that we used only male pigs, which could have an impact on the results because there are sex-related differences in clotting in humans [27], but it is female humans who clot better than males, so any sex difference bias should have worked against obtaining a favorable response in this study. Nonetheless, future studies are planned in which female and male swine are used.

Taken together, the current results strengthen the rationale for developing POSS-based visco-liquid hemostats as a new category of internal organ sealants capable of addressing the longstanding clinical gap in prehospital management of non-compressible thoracic hemorrhage. Future work should incorporate comparative studies against emerging commercial lung sealants and explore device-based delivery methods that enable rapid deployment in austere settings.

## Figures and Tables

**Table 1:** Mean systemic arterial pressure (MAP; mm Hg) and heart rate (HR, beats/min) recorded continuously and analyzed in 5 min bursts during pre-incisional control (15 min), and during 10 min in the polyhedral oligomeric silsesquioxane (POSS), POSS + chitin (PC, 10%), POSS + kaolin (PK, 22.5%), untreated control (UNT), and gauze + compression control (GC) treatment groups. No significant changes occurred in either variable for the duration of the experiment, nor were there any differences among the groups.

	PRE	POSS	PC	PK	UNT	GC
MAP (mm Hg)	87 ± 6	84 ± 6	85 ± 8	80 ± 6	86 ± 13	85 ± 13
HR (beats/min)	95 ± 3	100 ± 5	94 ± 5	97 ± 3	100 ± 5	98 ± 5

**Table 2:** Estimated mean differences in minutes-to-clot using Tobit regression with robust standard errors. Abbreviations: CI = confidence interval; UNT = untreated; GC = gauze + compression; POSS = polyhedral oligomeric silsesquioxane; PC = POSS + chitin (PC, 10%); PK = POSS + kaolin (PK, 22.5%). Tukey’s method was used to adjust the CIs and *p*-values for multiple comparisons.

Treatment Comparison	Mean Difference (95% CI)	*p*
UNT-GC	5.0 (−5.1 to 15.2)	0.60
UNT-PC	13.3 (5.5 to 21.0)	**<0.001**
UNT-PK	8.4 (1.7 to 15.0)	**0.008**
UNT-POSS	12.8 (4.2 to 21.4)	**0.001**
GC-PC	8.3 (0.6 to 15.9)	**0.03**
GC-PK	3.3 (−4.6 to 11.2)	0.73
GC-POSS	7.7 (2.5 to 12.9)	**0.001**
PC-PK	−4.9 (−11.1 to 1.2)	0.17
PC-POSS	−0.5 (−5.5 to 4.4)	1.0
PK-POSS	4.4 (−2.9 to 11.7)	0.41

## Data Availability

The original contributions presented in this study are included in the article. Further inquiries can be directed to the corresponding author.
